# Virtual reality tasks with eye tracking for mild spatial neglect assessment: a pilot study with acute stroke patients

**DOI:** 10.3389/fpsyg.2024.1319944

**Published:** 2024-01-29

**Authors:** Jenni Uimonen, Sanna Villarreal, Siiri Laari, Anne Arola, Petra Ijäs, Juha Salmi, Marja Hietanen

**Affiliations:** ^1^Department of Neuropsychology, Helsinki University Hospital and University of Helsinki, Helsinki, Finland; ^2^Department of Psychology and Logopedics, Faculty of Medicine, University of Helsinki, Helsinki, Finland; ^3^Department of Neurology, Helsinki University Hospital and University of Helsinki, Helsinki, Finland; ^4^Department of Neuroscience and Biomedical Engineering, Aalto University, Espoo, Finland

**Keywords:** visual neglect, extinction, immersive virtual environment, gaze tracking, gaze asymmetry, three-dimensional space, neuropsychological assessment

## Abstract

**Objective:**

Increasing evidence shows that traditional neuropsychological tests are insensitive for detecting mild unilateral spatial neglect (USN), lack ecological validity, and are unable to clarify USN in all different spatial domains. Here we present a new, fully immersive virtual reality (VR) task battery with integrated eye tracking for mild visual USN and extinction assessment in the acute state of stroke to overthrow these limitations.

**Methods:**

We included 11 right-sided stroke patients and 10 healthy controls aged 18−75 years. Three VR tasks named the Extinction, the Storage and the Shoot the target tasks were developed to assess USN. Furthermore, neuropsychological assessment examining various parts of cognitive functioning was conducted to measure general abilities. We compared VR and neuropsychological task performance in stroke patients – those with (USN+, *n* = 5) and without USN (USN−, *n* = 6) – to healthy controls (*n* = 10) and tentatively reported the usability of VR system in the acute state of stroke.

**Results:**

Patients had mostly mild neurological and USN symptoms. Nonetheless, we found several differences between the USN+ and healthy control groups in VR task performance. Compared to controls, USN+ patients showed visual extinction and asymmetry in gaze behavior and detection times in distinct spatial locations. Extinction was most evident in the extrapersonal space and delayed detection times on the extreme left and on the left upper parts. Also, USN+ patients needed more time to complete TMT A compared with USN− patients and TMT B compared with controls. VR system usability and acceptance were rated high; no relevant adverse effects occurred.

**Conclusion:**

New VR technology with eye tracking enables ecologically valid and objective assessment methods with various exact measures for mild USN and thus could potentially improve future clinical assessments.

## Introduction

Unilateral spatial neglect (USN) is a heterogenous syndrome comprising various attentional and spatial symptoms with adverse impacts on several daily functions, for example, reading and spatial navigation (Bosma et al., 2020). USN is characterized by a failure to orient, report, or respond to stimuli located on the opposite side of the brain lesion (Heilman et al., 2000). USN is more common after a right hemisphere stroke and therefore it is more often manifested on the left side of the perceptual space or body (Corbetta et al., 2005; Zebhauser et al., 2019). This spatial bias may occur together with an extinction, which is the inability to perceive a contralesional stimulus presented concurrently with an ipsilesional stimulus (Becker and Karnath, 2007), or deficits in non-lateralized attention, visuospatial working memory or processing speed (Bonato, 2012). Brief paper-and-pencil tests are used to assess USN, even though a large body of research has shown that their sensitivity is variable, and they are imprecise in detecting mild USN (Williams et al., 2021; Kaiser et al., 2022; Guilbert, 2023).

The use of computer-based methods has been suggested to enhance diagnostic accuracy (Bonato, 2012; Bonato et al., 2012) because they can improve sensitivity and psychometric properties of USN assessment (Giannakou et al., 2022). Computer-based assessment with large field of view and especially with a dual task have been reported to uncover contralesional visual attention deficits more precisely than traditional USN tests (Villarreal et al., 2020). However, computer-based applications have been criticized for having poor ecological validity because the tests typically do not resemble daily life situations (Ulm et al., 2013). Computer-based and paper-and-pencil tests are performed in a two-dimensional space and do not assess peripersonal (within arm’s reach) and extrapersonal space (beyond arm’s reach) simultaneously (Cavedoni et al., 2022). This would be important because USN can occur selectively in near or far space (Kerkhoff, 2001). It has been proposed that restrictions with traditional methods can be solved using virtual reality (VR) technology, which allows simulating a complex set of actions in a more realistic three-dimensional environment and creating a sense of presence in an experimentally controlled setting (Tsirlin et al., 2009; Pedroli et al., 2015; Ogourtsova et al., 2017).

Prior studies using cancellation, detection, navigation, or road crossing tasks have revealed that VR assessment can effectively detect USN, and correlations with VR and traditional USN tasks have been demonstrated (described in detail in reviews; Tsirlin et al., 2009; Pedroli et al., 2015; Ogourtsova et al., 2017; Cavedoni et al., 2022; Terruzzi et al., 2023). What has been largely missing, however, are VR studies assessing extinction in different spatial domains (see Fordell et al., 2011). Moreover, only a few USN studies utilize fully immersive VR with integrated eye-tracking cameras (Sugihara et al., 2016; Hougaard et al., 2021; Knoppe et al., 2022). This new technology allows collection of gaze coordinates and path, number of fixation points, saccade and fixation duration, and several other measures pinpointing processes reflecting visual spatial attention (Duchowski, 2002; Clay et al., 2019; Kaufmann et al., 2019; Merzon et al., 2022). Gaze-based responses enable task performance monitoring with millisecond level temporal precision (Hougaard et al., 2021) and search behavior evaluation without motor skills affecting the performance. Fully immersive VR studies with eye tracking and gaze behavior measures have demonstrated that patients with left USN may show orientation bias toward the ipsilesional side (Knoppe et al., 2022) and rightward deviation in gaze and head orientation (Hougaard et al., 2021). In some VR studies, higher task complexity has improved detection of USN deficits (Buxbaum et al., 2008; Ogourtsova et al., 2018a), but this issue has so far only sparsely been examined with VR eye-tracking methods in USN patients (Knoppe et al., 2022).

Previous VR studies have reported delayed contralesional detection times in USN patient groups when using Cartesian (Dvorkin et al., 2012; Ogourtsova et al., 2018b) or spherical coordinate system (Kim et al., 2021, Numao et al., 2021). Impaired contralesional visual information processing of USN patients has been associated with wider angles when using polar coordinates (Dvorkin et al., 2012). Nonetheless, detection times for targets in different spatial locations along the horizontal and vertical axis have not yet been mapped sufficiently in USN patients with eye-tracking VR methods. Moreover, many of the previous USN related VR studies have assessed patients in subacute or chronic states (typically 1−6 months post stroke), and some studies lacked the use of control groups and usability assessments concerning VR systems (see Pedroli et al., 2015; Ogourtsova et al., 2017; Cavedoni et al., 2022). Usability should be assessed with any novel VR-based platform because some head-mounted displays (HDM) and VR task implementations may expose to cybersickness and other adverse effects (Cavedoni et al., 2022). Performance of healthy individuals and stroke patients with and without USN should be examined (Terruzzi et al., 2023) and assessments performed also in the acute state of stroke when USN symptoms are most prominent and patients are less likely able to compensate USN (Bonato, 2012).

The primary aim of this pilot study was to introduce a new VR task battery for mild visual USN assessment and to compare the VR task performance of patients with and without USN to controls. The VR battery included three tasks evaluating different USN aspects: (1) The Extinction task for demonstrating visual extinction in peripersonal and extrapersonal space, (2) The Storage task to assess gaze behavior and detection times in different locations in environment resembling real life, and (3) The Shoot the target single and multiple task variants for clarifying contra- and ipsilesional deficits on distinct horizontal and vertical locations in time-limited conditions. The multiple task version was used to assess the effect of cognitive load in USN manifestation. In this pilot study, we also report preliminary findings on the feasibility and acceptance (i.e., realism, accessibility, possible side effects) of the VR tasks using a questionnaire created for the study. This is important before continuing to a larger study employing similar tasks as many of the previous studies have not examined these aspects. To our knowledge, this is one of the first VR studies to carry out the USN and extinction assessment in the acute state of stroke and to use a fully immersive HDM with an integrated eye-tracking camera and mainly gaze-based data.

## Materials and methods

### Participants

Eleven patients with right hemisphere stroke and 10 healthy controls matched with age, gender, and education were studied in Helsinki University Hospital’s acute neurological wards from 16.3.2021 – 19.1.2023. Patients were screened based on medical records and acute phase neurological evaluation (see neurological examination section below). Inclusion criteria were (1) first-ever diagnosed supratentorial right-sided ischaemic stroke, (2) age 18−75 years, and (3) native Finnish speaker. Exclusion criteria were (1) occipital or bilateral stroke, (2) any history of neurological or severe psychiatric disease known to affect cognition, (3) substance use disorders, (4) medically unstable condition, (5) primary problems with vision or hearing (other than myopia/hyperopia corrected with glasses), (6) hemianopia, (7) pacemaker or other medical device implemented in the body, or (8) infection in eyes, skin, or scalp, and (9) severe aphasia or other conditions significantly impairing cooperation. After systematic recruitment, 11 stroke patients met the criteria (age median, MD = 52, Interquartile Range, IQR = 14 years). Four eligible candidates refused to participate in the study, and five patients did not attend for logistical reasons, such as a rapid discharge to secondary care.

The patients were divided into USN (USN+; *n* = 5) and non-USN (USN−; *n* = 6) groups according to their performance in traditional USN assessment tests and clinical observation. In this study, USN was diagnosed if one or more of the four USN evaluation tests were below the cut-off, or if the patient had impaired spatial attention in daily life activities according to the Catherine Bergego Scale (CBS) self-evaluation form (Azouvi, 1996) or reports of USN in their medical record (Supplementary Table 1). The four used USN evaluation tests were the Bell Cancellation Test (Bells Test) (Gauthier et al., 1989) and three subtests of Behavioral Inattention Test (BIT) (Wilson et al., 1987). Reports of USN were based on acute state clinical examinations and observations made by other professionals such as an experienced neuropsychologist, a neurologist, an occupational therapist or physiotherapist (described in detail in the Supplementary Table 1). According to the cut-off criteria, two patients showed USN in the Bells test, one patient in the BIT line bisection test, and three patients in the CBS self-evaluation form. All of the USN+ patients had also reports of USN in their medical record. Although on an individual level specific traditional tests had the ability to differentiate specific participants, when the results of the traditional tests were pooled together, the effects were no longer observed (*p* > 0.05) (see Supplementary Table 1). Mild USN was characterized by a positive result on one to three USN tests and CBS self-evaluation score of 10 or below. Moderate USN was determined by a positive result on four USN tests or CBS self-evaluation score 11−20 (Azouvi et al., 2002, Ogourtsova et al., 2018b). Supplementary Table 1 shows that four of the USN+ patients had mild USN, and one patient had moderate USN.

A control group of 10 participants (age MD = 50, IQR = 23 years) recruited from healthy volunteers met all other inclusion and exclusion criteria except stroke. Table 1 shows the patients’ and controls’ characteristics. All participants provided written informed consent. This study was approved by Helsinki University Hospital’s Ethics Committee and completed in accordance with Helsinki Declaration.

**TABLE 1:** Demographics and neurological characteristics of stroke patients and controls.

Variables	USN+ (*n* = 5)	USN− (*n* = 6)	Controls (*n* = 10)
Age, years ^a^	45 (26)	53.5 (11)	50 (23)
Education, years ^a^	13 (4)	15.5 (5)	15.3 (5)
Gender, number of male/female	3/2	3/3	5/5
Handedness, number of right/left/ambidextrous	4/0/1	5/1/0	9/1/0
Vascular risk factors (reported by participants)			
Hypertension, *n* (%)	1 (20%)	2 (33%)	3 (30%)
Diabetes, *n* (%)	1 (20%)	1 (17%)	1 (10%)
Heart diseases, *n* (%)	2 (40%)	0	0
Days post-onset of stroke prior to study ^a^	10 (11)	10.5 (10)	
NIHSS, acute state ^a^, min-max	2 (7), 0−9	0 (2), 0−3	
mRS, acute state ^a^, min-max	2 (3), 1−4	1 (0), 1	
Barthel Index ^a^, min-max	100 (48), 35−100	100 (0), 100	
Imaging method, number of CT/MRI-verified	5/ 2	6/ 6	
Location of stroke			
Frontal lobe, *n*	0	3	
Parietal lobe, *n*	0	1	
Temporal lobe, *n*	2	0	
Basal Ganglia, *n*	1	1	
Other, *n*	0	0	
Several, *n*	2	1	
Vessel territory of stroke, number of ACA/MCA/PCA/several	0/ 3/ 0/ 2	2/ 3/ 1/ 0	
Type of stroke			
Partial anterior, *n*	5	5	
Total anterior, *n*	0	0	
Lacunar, *n*	0	1	
Posterior, *n*	0	0	
Stroke size, diameter in mm ^a^	50 (23)	12 (8)	
Thrombolysis, *n* (%)	1 (20%)	1 (17%)	

USN, Unilateral spatial neglect; USN+, Patients with USN; USN−, Patients without USN; C, Controls; NIHSS, National Institute of Health Stroke Scale; mRS, modified Ranking Scale; CT, computed tomography; MRI, magnetic resonance imaging; ACA, Anterior cerebral artery; MCA, Middle cerebral artery; PCA, Posterior cerebral artery.

^a^ Median (Interquartile range).

### Test protocol

Every patient completed the study in an acute state of stroke over one to two sessions (range between sessions 0−11 days, median 1 days). The study comprised a short neurological examination and a neuropsychological assessment that included traditional paper-and-pencil tasks, VR tasks and a structured interview. The VR task order was randomized to control for possible fatigue but the other tasks were performed in fixed order in the task battery. All testing material was presented facing the participant’s midline. The control participants were assessed in one session with the same neuropsychological methods. The test session duration was 90−120 min per participant.

### Neurological examination

A stroke neurologist assessed stroke severity during acute phase (range 24 h − 7 days) by the National Institutes of Health Stroke Scale (NIHSS) (Brott et al., 1989) and disability by the modified Ranking Scale (mRS) (Farrell et al., 1991). Stroke severity was defined on the basis of these NIHSS scores: mild (1 − 5 points), mild to moderately severe (5 − 14 points), severe (15 − 24 points), and very severe (over 25 points) (Brott et al., 1989). Visual fields were tested at arrival and during follow-up to exclude hemianopia. Neurologist evaluated the computed tomography (CT) and/or magnetic resonance imaging (MRI) results for the side, size, location and the vessel territory of infarct. The stroke type was classified by the Bamford classification system (Donnan et al., 2008). The patients’ basic functional status was assessed with the Barthel Index (B.I.) (Mahoney and Barthel, 1965). Table 1 shows the neurological information.

### Neuropsychological assessment

Various other neuropsychological tests were acquired. Processing speed was measured with time (s) to complete the Trail Making Test (TMT) part A and B (Reitan, 1958). Executive function was measured with a subtraction score (s) of the TMT forms B and A and attention with a number of errors in TMT A+B. Spatial short-term memory was examined with Spatial span total score from the Wechsler Memory Scale (WMS-III) (Wechsler, 1997; Wechsler, 2008). Visual perceptual function was assessed with the total score from the Poppelreuter overlapping figure test (Christensen, 1974) and motor skills with the Finger Tapping test score in the dominant hand (Lezak et al., 2004). General cognition was screened with the Montreal Cognitive Assessment Scale (MoCA) (Nasreddine et al., 2005). In MoCA, scores between 18−25 indicate mild cognitive impairment and scores under 18 moderate/severe cognitive impairment.

### Virtual reality tasks

#### The Extinction task

**FIGURE 1:**
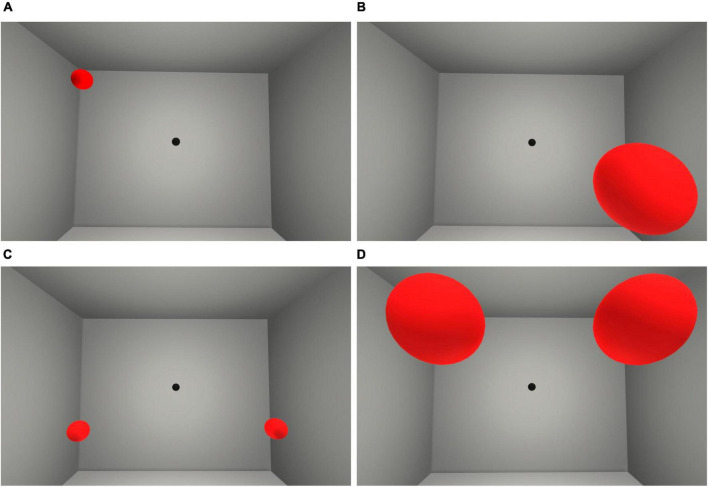
Visualization of the Extinction task from the participants’ view. The top row shows unilateral targets on the upper left distal **(A)** and the lower right proximal visual space **(B)**. The bottom row shows bilateral targets on the lower distal **(C)** and the upper proximal visual space **(D)**. Participants were instructed to maintain their gaze at fixation point (a black ball) at the center of the view to control eye fixation and to decrease the possibility of the participant of focusing only on the lateralized targets. Test was paused if participants looked away from the fixation point.

The basic view was an empty room where target(s) appeared symmetrically with respect to a vertical midline in the HDM in eight quadrants: upper left/right distal, upper left/right proximal, lower left/right distal, lower left/right proximal (Figure 1). As the targets were presented only for 100−300 ms, recording the responses based on gaze would have been problematic and the results would have been unreliable. Instead, participants were instructed to verbally report the position of the target(s) after detecting them. Some stroke patients may have a hemiparesis or hemiplegia, so the experimenter was holding VR controllers in both hands and verified participants’ responses [location of the target(s) in the “left,” “right,” or “both” side(s)] with the controller. Supplementary Table 2 presents detailed information.

#### The Storage task

**FIGURE 2:**
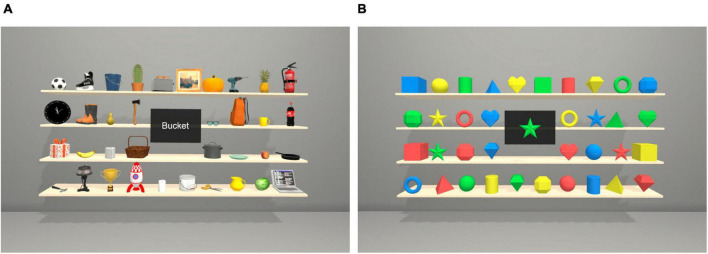
Visualization of the Storage task with objects **(A)** and figures **(B)** from the participants’ view. The target’s name or picture appeared one at a time in the black square in the middle of the storage shelf. In the Storage task with objects, participants also heard the name of the target through the headset.

The scene contained a symmetrical room displaying a storage shelf located in front and 3 m away from the participants (Figure 2). The shelves held either different objects used in everyday life or figures in different colors that could be seen constantly. The participants were instructed to search for a specific target from the storage shelf as quickly as possible and choose the target by maintaining their gaze to it for 2.2 s. Supplementary Table 2 presents the task’s technical parameters.

#### The Shoot the target task

**FIGURE 3:**
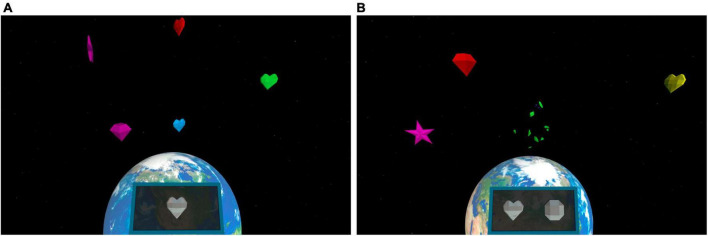
The VR scene used in the Shoot the target task. The participants were instructed to search for one target in the single task **(A)** and for two targets at a time in the multiple task **(B)**. Correct target type(s) could be seen in the black square in the middle lower part of the surrounding visual space, which changed every 30 s.

The viewer was located in a space station, and the targets and distractors appeared randomly at 3−4 meters distance from participants in four quadrants (lower left/right, upper left/right) (Figure 3). The participants were instructed to search for certain types of targets and choose the targets by orienting their gaze to them. See Supplementary Table 2 for detailed information.

### VR apparatus

The Peili Vision Company conducted the implementation of the tasks. A Pico Neo 2 Eye head-mounted display (HMD) (with a resolution of 1920 × 2160 pixels per eye, 75 Hz refresh rate, and 101-degree field of view) and its hand controller were used to perform the tasks. Gaze position was recorded with Tobii 90 Hz eye tracker with 0.5 degree stated system accuracy integrated into the VR headset. Tasks were launched and observed by the experimenter via Samsung Galaxy Tab S3 tablet. The participants wore an HMD while seated in a chair. They performed three distinct visual attention tasks with a short, two-minute break between the tasks. The scene was viewer centered in all the tasks regardless of head orientation. Calibration and validation of the eye-tracking camera was performed before every session and after the short break to ensure assessment accuracy. No difference was observed between the groups for either calibration measurement [measurement 1, H(2) = 2.310, *p* = 0.315; measurement 2, H(2) = 1.036, *p* = 0.596]. Verbal guidance and a short training preceded the VR tasks. All participants were novices to the VR tasks. After performing the tasks, all participants were asked to complete a questionnaire on their prior VR experience and on the VR tasks’ usability and possible adverse effects. The questionnaire was inspired by previous VR studies (Fordell et al., 2011, Gil-Gomez et al., 2017).

### Statistical methods

The statistical analyses were conducted with IBM SPSS Statistics software, V.28.0 (International Business Machines Corporation, Armonk, New York, USA). Demographics, clinical and neuropsychological data were analyzed using nonparametric methods due to the skewed distribution of the variables and small sample size. Analyses were performed using the Mann-Whitney U or Kruskal-Wallis Tests (U/χ2) for continuous variables and the Pearson’s Chi-Square Test (χ2) or Fisher’s Exact Test for categorical variables. Bonferroni correction was used to correct for the multiple comparisons. Effect sizes were calculated by computing eta squared (η2) for the Kruskal-Wallis test and r for the Mann-Whitney *U* test. For statistically significant group differences, we used Cohen’s descriptions for η2 (small effect: 0.01, medium effect: 0.06, and large effect; 0.14) and for r (small effect: 0.1, medium effect: 0.3, and large effect; 0.5) (Cohen, 1988). Finally, we examined the associations between VR tasks and traditional paper-and-pencil USN evaluation tests by using Kendall’s rank correlation coefficient. The statistical significance level was set at *p* <0 .05.

## Results

### Demographics and neurological examination

Table 1 presents the participants’ demographics and neurological information. There were no differences in gender (*p* = 1.000, Fisher’s Exact Test), age [(*H*(2) = 1.277, *p* = 0.528] or years of education [*H*(2) = 1.151, *p* = 0.562] between the three groups (USN+ patients, USN− patients, and controls). Most of the participants were right-handed, and the handedness did not differ between the three groups (*p* = 0.662, Fisher’s Exact Test). One USN+ patient reported to be ambidextrous, but used the right hand in the test situation since patient estimated the right hand to be more dominant than the left hand. The only left-handed stroke patient in our study had no hemiplegia at the time of the study according to NIHSS (0 points) and finger tapping test (51/10 s). According to NIHSS, five patients had no remaining neurological symptoms, four patients had mild neurological symptoms, and two patients had mild to moderate neurological symptoms. The USN+ (MD = 2, IQR = 7) and USN− (MD = 0, IQR = 2) groups did not differ in NIHSS scores (*U* = 6.500, *p* = 0.103).

### Neuropsychological assessment

Table 2 presents neuropsychological task performance. USN+ patients needed more time to complete TMT A compared to USN− patients and TMT B compared to healthy controls. USN+ patients’ scores in MoCA were lower than controls’. Seven patients’ (five USN+, 2 USN−) MoCA scores were under 26 (range 19−25 indicating mild cognitive impairment), whereas none of the controls scored under 26. No group differences in other neuropsychological variables were detected.

**TABLE 2:** Task performance in neuropsychological tests.

Variables	USN+ (*n* = 5)	USN− (*n* = 6)	Controls (*n* = 11)	*p*	Effect size^d^
TMT A time (s) ^a, b^	50 (41.5)	22.5 (12)	28.5 (15.8)	0.011	η2 = 0.390***
*Post-hoc* comparisons ^c^					
USN+ vs. USN−				0.03	*r* = 0.772***
USN− vs. C				0.429	
USN+ vs. C				0.060	
TMT B time (s) ^a, b^	123 (96.5)	64.5 (42)	70 (44)	0.027	η2 = 0.289***
*Post-hoc* comparisons ^c^					
USN+ vs. USN−				0.084	
USN− vs. C				1.000	
USN+ vs. C				0.042	*r* = 0.632***
TMT subtraction score B-A (s) ^a, b^	91 (97)	45 (40.5)	39 (28.8)	0.440	
TMT A+B mistakes (number) ^a, b^	1 (5.5)	0 (0)	0 (1)	0.080	
Spatial span total score ^a, b^	16 (4)	21.5 (8)	17.5 (7)	0.059	
Poppelreuter score (1−14) ^a, b^	14 (1)	14 (0)	14 (0)	0.202	
Tapping test score dominant hand ^a, b^	60 (19)	52 (7.3)	54.5 (12)	0.437	
MoCA score (0−30) ^a, b^	24 (4)	26.5 (5)	27 (2)	0.014	η2 = −0.360***
*Post-hoc* comparisons ^c^					
USN+ vs. USN−				0.357	
USN− vs. C				1.000	
USN+ vs. C				0.006	*r* = 0.807***

USN, Unilateral spatial neglect; USN+, Patients with USN; USN−, Patients without USN; C, Controls; TMT, Trail Making Test; MoCA, Montreal Cognitive Assessment Scale.

^a^ Median (Interquartile range).

^b^ *p* values were calculated by Kruskal-Wallis test (χ2).

^c^ Mann-Whitney *U*-test was used for multiple pairwise comparisons, *p* values adjusted by the Bonferroni correction.

^d^ Effect sizes according to Cohen, 1988: η^2^ = small > 0.01, medium > 0.06,

***large > 0.14 and r = small > 0.1, medium > 0.3, ***large > 0.5.

### Virtual reality tasks

#### The Extinction task

Table 3 presents between-group comparisons. USN+ patients detected a lower number of bilateral targets than controls (USN+ MD = 12, IQR = 10; Controls MD = 16, IQR = 0). Looking more closely, USN+ patients had more omissions specifically on the left side for bilaterally appearing targets (USN+ MD = 4, IQR = 9; Controls MD = 0, IQR = 0). Omissions were also examined at the individual level due to the study’s pilot nature. We learned that three of the five USN+ patients had omissions in the task when targets were presented bilaterally (omission numbers per patient varied between 4−13). Targets in the task appeared in dimensions left/right, upper/lower, and proximal/distal. Left-side omissions on bilaterally presented targets were mostly positioned in the upper (68%) and distal (77%) parts of the visual field when considered on the individual level.

**TABLE 3:** Task performance in the Extinction task.

Variables	USN+ (*n* = 5)	USN− (*n* = 6)	Controls (*n* = 10)	χ 2/*U*	df	*p*	Effect size^d^
Correct targets total (0−48) ^a, b^	41 (12)	48 (0)	48 (0)	7.840	2	0.020	η2 = 0.324***
*Post-hoc* comparisons ^c^							
USN+ vs. USN−				6.000		0.111	
USN− vs. C				27.000		1.000	
USN+ vs. C				11.000		0.084	
Correct unilateral targets left (0−16) ^a, b^	16 (4)	16 (0)	16 (0)	4.103	2	0.129	
Correct unilateral targets right (0−16) ^a, b^	16 (0)	16 (0)	16 (0)	0.000	2	1.000	
Correct bilateral targets (0−16) ^a, b^, min - max	12 (10), 3−16	16 (0), 16	16 (0), 16	10.592	2	0.005	η2 = 0.477***
*Post-hoc* comparisons ^c^							
USN+ vs. USN−				6.000		0.111	
USN− vs. C				30.000		1.000	
USN+ vs. C				10.000		0.027	*r* = 0.678***
Bilateral targets, left omissions (0−16) ^a, b^, min - max	4 (9), 4−13	0 (0), 0	0 (0), 0	10.592	2	0.005	η2 = 0.477***
*Post-hoc* comparisons ^c^							
USN+ vs. USN−				6.000		0.111	
USN− vs. C				30.000		1.000	
USN+ vs. C				10.000		0.027	*r* = 0.678***
Bilateral targets, right omissions (0−16) ^a, b^	0 (0)	0 (0)	0 (0)	0.000	2	1.000	
Bilateral targets, right & left omissions (0−16) ^a, b^	0 (1)	0 (0)	0 (0)	3.200	2	0.202	

USN, Unilateral spatial neglect; USN+, Patients with USN; USN−, Patients without USN; C, Controls.

^a^ Median (Interquartile range).

^b^ *p* values were calculated by Kruskal-Wallis test (χ2).

^c^ Mann-Whitney *U*-test was used for multiple pairwise comparisons, *p* values adjusted by the Bonferroni correction.

^d^ Effect sizes according to Cohen, 1988: η^2^ = small > 0.01, medium > 0.06,

***large > 0.14 and r = small > 0.1, medium > 0.3, ***large > 0.5.

#### The Storage task with objects

Group differences were observed in six measures of the Storage task with objects (Tables 4, 5). USN+ patients’ total search time was longer than USN− patients’ and controls’. USN+ patients’ detection times on the left were longer compared to controls’, especially in the extreme left compared with USN– patients and controls. Also, gaze duration (%) was shorter on the left and longer on the right for USN+ patients than controls. The gaze asymmetry score showed that USN+ patients had less and controls had more gaze on the left. No differences between the groups in other variables were observed after Bonferroni corrections.

**TABLE 4:** Detection times in the Storage subtask with objects.

Variables	USN+ (*n* = 5)	USN– (*n* = 6)	Controls (*n* = 10)	χ 2/*U*	df	*p*	Effect size^d^
Total search time (s) ^a, b^	173 (78)	120 (28)	138 (25)	8.921	2	0.012	η2 = 0.385***
*Post-hoc* comparisons ^c^							
USN+ vs. USN−				1.000		0.033	*r* = 0.771***
USN− vs. C				19.000		0.699	
USN+ vs. C				5.000		0.042	*r* = 0.632***
Detection time total (ms) ^a, b^	3504 (2976)	1438 (1301)	1840 (1324)	7.161	2	0.028	η2 = 0.287***
*Post-hoc* comparisons ^c^							
USN+ vs. USN–				2.000		0.054	
USN– vs. C				22.000		1.000	
USN+ vs. C				7.000		0.081	
Detection time left (ms) ^a, b^	3673 (3827)	1831 (2161)	1663 (1794)	6.591	2	0.037	η2 = 0.255***
*Post-hoc* comparisons ^c^							
USN+ vs. USN−				4.000		0.135	
USN− vs. C				27.000		1.000	
USN+ vs. C				5.000		0.042	*r* = 0.632***
Detection time right (ms) ^a, b^	2630 (2419)	1415 (871)	2041 (672)	5.638	2	0.060	
Detection time extreme left (ms) ^a, b^	5295 (4041)	1987 (1951)	1753 (1591)	9.339	2	0.009	η2 = 0.408***
*Post-hoc* comparisons ^c^							
USN+ vs. USN−				1.000		0.033	*r* = 0.771***
USN− vs. C				29.000		1.000	
USN+ vs. C				2.000		0.015	*r* = 0.727***
Detection time middle left (ms) ^a, b^	2712 (3914)	1536 (2105)	1641 (2034)	3.397	2	0.183	
Detection time extreme right (ms) ^a, b^	1812 (917)	1322 (590)	1586 (620)	2.158	2	0.340	
Detection time middle right (ms) ^a, b^	3475 (3961)	1478 (938)	2121 (101)	5.243	2	0.073	
Detection time upper parts (ms) ^a, b^	4046 (3145)	1523 (1190)	1780 (1041)	6.365	2	0.041	η2 = 0.243***
*Post-hoc* comparisons ^c^							
USN+ vs. USN−				4.000		0.135	
USN− vs. C				24.000		1.000	
USN+ vs. C				6.000		0.060	
Detection time lower parts (ms) ^a, b^	3021 (3389)	1372 (1266)	1876 (1203)	5.648	2	0.059	
Detection time left upper parts (ms) ^a, b^	4343 (5654)	1924 (2235)	1565 (1400)	4.613	2	0.100	
Detection time left lower parts (ms) ^a, b^	4365 (3123)	1633 (2068)	1356 (1967)	6.189	2	0.045	η2 = 0.233***
*Post-hoc* comparisons^c^							
USN+ vs. USN−				3.000		0.084	
USN− vs. C				28.000		1.000	
USN+ vs. C				7.000		0.081	
Detection time right upper parts (ms) ^a, b^	2507 (2312)	1305 (859)	1703 (702)	3.958	2	0.138	
Detection time right lower parts (ms) ^a, b^	2120 (4764)	1547 (604)	2203 (1061)	4.347	2	0.114	

USN, Unilateral spatial neglect; USN+, Patients with USN; USN−, Patients without USN; C, Controls.

^a^Median (Interquartile range).

^b^ *p* values were calculated by Kruskal-Wallis test (χ2).

^c^ Mann-Whitney *U*-test was used for multiple pairwise comparisons, *p* values adjusted by the Bonferroni correction.

^d^ Effect sizes according to Cohen, 1988: η^2^ = small > 0.01, medium > 0.06,

***large > 0.14 and r = small > 0.1, medium > 0.3, ***large > 0.5.

**TABLE 5:** Task performance in the Storage subtask with objects.

Variables	USN+ (*n* = 5)	USN− (*n* = 6)	Controls (*n* = 10)	χ 2/*U*	df	*p*	Effect size^d^
Omissions total ^a, b^	1 (6)	0 (0)	0 (0)	3.732	2	0.155	
Omissions left ^a, b^	0 (4)	0 (0)	0 (0)	2.193	2	0.334	
Omissions right ^a, b^	1 (2)	0 (0)	0 (0)	5.647	2	0.059	
Incorrect target selection ^a, b^	0 (2)	0 (0)	0 (1)	2.681	2	0.262	
Total head movement when playing (m) ^a,b^	3.3 (5)	3 (3.1)	3.2 (1.7)	1.112	2	0.571	
Gaze asymmetry score left/right ^a,b^	0.83 (0.31)	1.01 (0.4)	1.27 (0.3)	9.26	2	0.010	η2 = 0.403***
*Post-hoc* comparisons ^c^							
USN+ vs. USN−				7.000		0.432	
USN− vs. C				12.000		0.153	
USN+ vs. C				3.000		0.021	*r* = 0.696***
Gaze duration in left (%) ^a, b^	42.2 (9.2)	48.3 (8.8)	53.8 (5.9)	10.025	2	0.007	η2 = 0.446***
*Post-hoc* comparisons ^c^							
USN+ vs. USN−				5.000		0.204	
USN− vs. C				13.000		0.195	
USN+ vs. C				2.000		0.015	*r* = 0.727***
Gaze duration in right (%) ^a, b^	49.7 (8.1)	47.3 (9.2)	42.3 (5.8)	7.979	2	0.019	η2 = 0.332***
*Post-hoc* comparisons ^c^							
USN+ vs. USN−				8.000		0.603	
USN− vs. C				14.000		0.249	
USN+ vs. C				4.000		0.030	*r* = 0.664***
Gaze duration in extreme left (%) ^a, b^	15.5 (6)	20.6 (3.3)	19.1 (5.9)	6.685	2	0.035	η2 = 0.260***
*Post-hoc* comparisons ^c^							
USN+ vs. USN−				2.000		0.054	
USN− vs. C				27.000		1.000	
USN+ vs. C				7.000		0.081	
Gaze duration in middle left (%) ^a, b^	28.9 (4.3)	28.4 (7.1)	32.1 (8.3)	6.631	2	0.036	η2 = 0.257***
*Post-hoc* comparisons ^c^							
USN+ vs. USN−				12.000		1.000	
USN− vs. C				12.000		0.153	
USN+ vs. C				7.000		0.081	
Gaze duration in middle right (%) ^a, b^	28.5 (8.4)	29 (7.3)	26.1 (7.2)	2.626	2	0.269	
Gaze duration in extreme right (%) ^a, b^	21.2 (6.9)	18.5 (3.6)	18.4 (4)	5.139	2	0.077	

USN, Unilateral spatial neglect; USN+, Patients with USN; USN−, Patients without USN; C, Controls.

^a^ Median (Interquartile range).

^b^ *p* values were calculated by Kruskal-Wallis test (χ2).

^c^ Mann-Whitney *U*-test was used for multiple pairwise comparisons, *p* values adjusted by the Bonferroni correction.

^d^ Effect sizes according to Cohen, 1988: η^2^ = small > 0.01, medium > 0.06,

***large > 0.14 and r = small > 0.1, medium > 0.3, ***large > 0.5.

#### The Storage task with figures

Supplementary Tables 3, 4 present between-group comparisons. USN+ patients’ total search time, total detection time, detection time in left, and detection time in extreme left were longer compared with USN− patients’ and controls’. USN+ patients’ detection time in the left lower parts was longer compared with USN− patients’. USN+ patients’ detection time in upper parts, left upper parts, and left lower parts were longer and gaze duration on the left (%) was shorter when compared with controls. There was no difference between the groups in analyses concerning omissions, incorrect target selection, or other gaze variables.

#### The Shoot the target single task

Tables 6, 7 present between-groups comparisons. USN+ patients’ total search time was longer than USN− patients’ and controls’. Also, target detection time was longer for USN+ patients than controls, likewise detection times in most of the different locations. However, there was no detection time difference between the groups on the right lower quadrant. USN+ patients’ total score left was lower than controls’. There were no differences between the groups in total score, total score right or incorrect target selection after Bonferroni corrections.

**TABLE 6:** Detection times in the Shoot the target single task.

Variables	USN+ (*n* = 5)	USN− (*n* = 6)	Controls (*n* = 10)	χ 2/*U*	df	*p*	Effect size^d^
Total search time (s) ^a, b^	122 (6)	111 (9)	109 (11)	10.573	2	0.005	η2 = 0.476***
*Post-hoc* comparisons ^c^							
USN+ vs. USN−				1.000		0.033	*r* = 0.771***
USN− vs. C				20.000		0.834	
USN+ vs. C				1.000		0.009	*r* = 0.759***
Detection time total (ms) ^a, b^	3030 (1065)	1990 (823)	1535 (770)	10.852	2	0.004	η2 = 0.492***
*Post-hoc* comparisons ^c^							
USN+ vs. USN−				2.000		0.054	
USN− vs. C				13.500		0.219	
USN+ vs. C				2.000		0.015	*r* = 0.728***
Detection time left (ms) ^a, b^	3660 (1025)	1840 (958)	1570 (800)	10.390	2	0.006	η2 = 0.466***
*Post-hoc* comparisons ^c^							
USN+ vs. USN−				3.000		0.084	
USN− vs. C				14.000		0.249	
USN+ vs. C				2.000		0.015	*r* = 0.728***
Detection time right (ms) ^a, b^	2570 (1265)	2065 (608)	1490 (615)	7.716	2	0.021	η2 = 0.318***
*Post-hoc* comparisons ^c^							
USN+ vs. USN−				7.000		0.432	
USN− vs. C				14.000		0.249	
USN+ vs. C				5.000		0.042	*r* = 0.632***
Detection time upper quadrants (ms) ^a, b^	3250 (1320)	2010 (810)	1465 (700)	11.802	2	0.003	η2 = 0.545***
*Post-hoc* comparisons ^c^							
USN+ vs. USN−				2.000		0.054	
USN− vs. C				12.000		0.153	
USN+ vs. C				1.000		0.009	*r* = 0.759***
Detection time lower quadrants (ms) ^a, b^	2240 (1810)	2020 (780)	1515 (830)	6.123	2	0.047	η2 = 0.229***
*Post-hoc* comparisons ^c^							
USN+ vs. USN−				8.000		0.600	
USN− vs. C				16.000		0.387	
USN+ vs. C				7.000		0.081	
Detection time upper left (ms) ^a, b^	3290 (1650)	1885 (1040)	1550 (555)	11.390	2	0.003	η2 = 0.522***
*Post-hoc* comparisons ^c^							
USN+ vs. USN−				2.500		0.066	
USN− vs. C				15.500		0.348	
USN+ vs. C				0.000		0.006	*r* = 0.791***
Detection time lower left (ms) ^a^ ^b^	2800 (1715)	2080 (1293)	1640 (973)	7.102	2	0.029	η2 = 0.283***
*Post-hoc* comparisons ^c^							
USN+ vs. USN−				5.000		0.204	
USN− vs. C				21.000		0.987	
USN+ vs. C				4.5000		0.036	*r* = 0.649***
Detection time upper right (ms) ^a, b^	3150 (1135)	2135 (690)	1500 (573)	10.324	2	0.006	η2 = 0.462***
*Post-hoc* comparisons ^c^							
USN+ vs. USN−				4.000		0.135	
USN− vs. C				13.000		0.195	
USN+ vs. C				2.000		0.015	*r* = 0.728**
Detection time lower right (ms) ^a,b^	1600 (2050)	2085 (713)	1445 (760)	2.786	2	0.248	

USN, Unilateral spatial neglect; USN+, Patients with USN; USN−, Patients without USN; C, Controls.

^a^ Median (Interquartile range).

^b^ *p* values were calculated by Kruskal-Wallis test (χ2).

^c^ Mann-Whitney *U*-test was used for multiple pairwise comparisons, *p* values adjusted by the Bonferroni correction.

^d^ Effect sizes according to Cohen, 1988: η^2^ = small > 0.01,

**medium > 0.06,

***large > 0.14 and r = small > 0.1, ** medium > 0.3, ***large > 0.5.

**TABLE 7:** Task performance in the Shoot the target single task.

Variables	USN+ (*n* = 5)	USN− (*n* = 6)	Controls (*n* = 10)	χ 2/*U*	df	*p*	Effect size^d^
Total score ^a, b^	0.82 (0.23)	0.97 (0.03)	0.98 (0.3)	5.228	2	0.073	
Total score left ^a, b^	0.83 (0.19)	0.98 (0.4)	1 (0.04)	6.908	2	0.032	η2 = 0.273***
*Post-hoc* comparisons ^c^							
USN+ vs. USN−				4.500		0.147	
USN− vs. C				25.500		1.000	
USN+ vs. C				6.500		0.042	*r* = 0.637**
Total score right ^a, b^	0.80 (0.27)	0.95 (0.02)	0.98 (0.06)	5.131	2	0.077	
Incorrect target selection ^a, b^	1 (3)	0 (0)	1 (1)	6.415	2	0.040	η2 = 0.245***
*Post-hoc* comparisons ^c^							
USN+ vs. USN−				4.500		0.105	
USN− vs. C				14.000		0.138	
USN+ vs. C				15.500		0.552	

USN, Unilateral spatial neglect; USN+, Patients with USN; USN−, Patients without USN; C, Controls.

^a^ Median (Interquartile range).

^b^ *p* values were calculated by Kruskal-Wallis test (χ2).

^c^ Mann-Whitney *U*-test was used for multiple pairwise comparisons, *p* values adjusted by the Bonferroni correction.

^d^ Effect sizes according to Cohen, 1988: η^2^ = small > 0.01,

**medium > 0.06,

***large > 0.14 and r = small > 0.1, ** medium > 0.3, ***large > 0.5.

#### The Shoot the target multiple task

Supplementary Tables 5, 6 present between-group comparisons. USN+ patients’ detection times were longer than controls’ on the left quadrants, on the upper quadrants, and on the left upper quadrant. The total score was lower for USN+ patients than controls. No differences between the groups for total search time, detection times on other quadrants, total score for the left or right, or incorrect target selection were seen after Bonferroni corrections.

### Correlations between USN evaluation tests and VR task battery

Supplementary Table 7 present the correlation coefficients between the traditional paper-and-pencil USN evaluation tests and VR tasks variables. In this analysis, we included most of VR task variables that showed significant differences in prior between-group comparisons (Tables 3–7, Supplementary Tables 3−6). We excluded variables from the Storage task with figures, since these two Storage tasks are similar. Instead, considering that many VR task variables are based on response time (e.g., time from target appearance to detection in different locations or total search time) we included BIT star cancellation time to correlation analysis. We found some significant (*p* < 0.05), but mostly low to moderate (*r* = 0.35−0.59) correlations between traditional USN evaluation tests and VR task variables. The highest correlations (*r* = 0.592) were between scores in the BIT subtasks and VR Extinction task. Over 50 % of the VR task variables correlated with amount of left-side omissions in the Bells test and performance time (s) in the BIT star cancellation test, while only VR Extinction task scores correlated with BIT line bisection test score and the number of correct targets in the BIT star cancellation test. Out of the single VR-task variables, correct responses for bilateral targets and left-side omissions on bilaterally presented targets in the VR Extinction task and objects gaze asymmetry score in the VR Storage task were correlated with 3/5 of the traditional USN evaluation tests.

### Prior VR experience

Over half of the participants (52%) reported that they had used VR goggles before. There was no difference in previous VR experience (*p* = 0.288, Fisher’s Exact Test) between the groups (USN+ patients, USN− patients, and controls). Nevertheless, to further examine if there was an effect of previous VR experience on VR task performance, we compared all participants’ (*n* = 21) performance depending on whether they have previous VR experience (*n* = 10) or not (*n* = 11). We did not find any differences in VR task performance between the groups in the 29 VR variables that were significant in prior analyses comparing USN+ patients, USN− patients and healthy controls’ task performance (see Tables 3−7, Supplementary Tables 3−6). As the analysis pooling the two groups (USN+ and USN− patients) together could possibly be inflated by the group differences, we repeated this VR exposure analysis in the sample of healthy participants. Out of these 10 participants six had previous VR experience and four did not. This analysis did not reveal any differences between the participants that had previous VR experience and the participants who did not.

### Adverse effects and VR tasks’ usability

Participants filled a questionnaire about adverse effects and the feasibility and acceptance of the VR tasks (Table 8). Participants did not report actual adverse effects, but one of the participants described that the VR display was too bright. Almost all participants (95%) understood the instructions for the VR tasks and felt that the VR device was comfortable and easy to use. All participants reported they felt focused, but three participants (14%) reported tiredness while playing. Almost all participants (91−93 %) felt that the VR environment was realistic and enjoyed the experience with the system.

**TABLE 8:** Participants’ responses to the questions regarding adverse effects, feasibility, acceptance, and usability of VR.

Questions	Yes	No
Adverse effects		
1. Did you feel nausea while playing?	0	21
2. Did you feel dizziness while playing?	0	21
3. Did you notice any seizure kind of symptoms while playing?	0	21
4. Did you notice any eye or muscle jerking while playing?	0	21
5. Did you feel fainting while playing?	0	21
6. Did you notice some other side effects while playing?	1*	20
Feasibility, acceptance, and usability		
7. Did you feel comfort to use VR?	20	1
8. Did you find the devices of the system easy to use?	20	1
9. Did you understand the instructions in the VR tasks?	20	1
10. Could you focus in the tasks?	21	0
11. Were you tired while playing?	3	18
12. Did you have the perception of the environment as being a realistic?	19	2
13. Did you enjoy your experience with the system?	20	1

*One of the participants reported that the VR display was too bright.

## Discussion

This pilot study introduces a new VR task battery for mild visual USN assessment that was developed to detect extinction and other USN related contralesional deficits, such as gaze asymmetry and delayed detection times in distinct spatial locations. Our preliminary results suggest that the VR task battery was able to differentiate USN+ patients from controls and reveal mild USN symptoms on a group level in the acute state of stroke. Regarding our primary aim, we demonstrated extinction in some of the USN+ patients, that was more evident in the distal (i.e., extrapersonal) than proximal (i.e., peripersonal) space. USN+ patients had also asymmetry in gaze behavior and were slower to detect targets on the left side in the VR tasks compared to controls, especially on the extreme left and on the left upper quadrants. USN+ patients showed both contralesional and non-lateralized attention deficits in the Shoot the target task. Concerning our second research question, the participants reported the VR experience enjoyable, user-friendly, and resulted no relevant adverse effects. Altogether, the results indicated that using VR with eye tracking may provide detailed information on mild USN and identify the specific area of neglect in different spatial locations.

One of the main finding was, that USN+ patients detected fewer bilateral targets and had more omissions on the left side when targets were presented simultaneously in the VR Extinction task. Only USN+ patients showed this visual extinction. Fordell et al. (2011) similarly showed that VR extinction task presented in peripersonal space correctly identified patients with USN. Previous studies have suggested that extinction can be one of the USN sub-symptoms or co-occurs with it (Driver and Vuilleumier, 2001; Brozzoli et al., 2006; Becker and Karnath, 2007), or may be a residual manifestation of USN in a chronic phase of recovery (Bonato, 2012; Andres et al., 2019). Interestingly, we found left-side omissions on bilaterally presented targets mostly in the upper (68%) and distal (77%) parts of the visual field when considered on the individual level. To our knowledge, extinction in different spatial domains, including radial, horizontal and vertical targets, has not been previously examined in stroke patients. However, a recent VR-based case study with unilateral targets showed similarly USN patient recognizing targets from larger visual angles in the near space than in the far space, and the angle of recognition tended to increase when the target’s height decreased (Yasuda et al., 2020). Our preliminary results, together with Yasuda et al. (2020) prior finding, imply that visual extinction, and USN in general, should be assessed in multiple spatial domains. Identifying USN subtypes in the early stroke state may help clinicians to improve patients’ awareness of the particular risks associated with specific tasks in their home environment or outdoors (Williams et al., 2021). Our new extinction task for VR provides several advantages in this regard, and could be a valid supplementary tool for neuropsychological assessment of USN.

We also discovered that USN+ patients’ gaze behavior differed from controls’ on the Storage task, especially in ecological scene with objects: USN+ patients’ gaze was less focused on the left and more focused on the right side than the controls’. Fully immersive VR studies with free exploration tasks have shown that gaze asymmetry (Hougaard et al., 2021) and the orientation bias toward the ipsilesional side are sensitive USN measures (Knoppe et al., 2022). Prior studies with eye tracking and non-immersive VR tasks (Cazzoli et al., 2016) or computerized tasks (Machner et al., 2018) have similarly reported a rightward bias in gaze patterns and eye movements in USN patients. Interestingly in our study, healthy controls’ gaze was more focused on the left side than on the right side. This possible pseudoneglect effect, that is, a natural tendency of allocating more spatial attention to the left side in healthy controls (Bowers and Heilman, 1980), has also been described in prior studies (Thomas et al., 2014; Perez-Marcos et al., 2023). Based on our findings, assessments with VR and eye-tracking measures could be a useful and objective tool to evaluate how USN patients perceive and explore virtual environments that simulate real-world scenarios. This could be further examined with tasks allowing participants to freely move and interact with a complex environment, such as a virtual homes or supermarkets (Faria et al., 2016; Glize et al., 2017; Seesjarvi et al., 2022).

Our results are aligned with previous VR and non-VR studies that have associated right hemisphere damage and contralesional USN with slow processing speed (Gerritsen et al., 2003; Bonato, 2012; Nurmi et al., 2018) and delayed reaction times both generally (van Kessel et al., 2010; Ogourtsova et al., 2018b; Kim et al., 2021) and contralesionally (Deouell et al., 2005; Rengachary et al., 2009; Kim et al., 2010; Aravind and Lamontagne, 2014; Ogourtsova et al., 2018b; Numao et al., 2021). USN+ patients completed TMT A slower than USN− patients and TMT B slower than controls in the present study. USN+ patients had also longer detection times than controls in two of four VR tasks and they needed more time to detect contralesional targets than controls in all four VR tasks. This was evident especially for targets appearing at extreme left locations in the Storage task with objects and figures when USN+ patients were compared with USN− patients and controls. Similarly, recent VR study has demonstrated increased reaction times when proceeding from the ipsilesional field toward the midline and into the contralesional side (Ogourtsova et al., 2018a). We also showed that USN+ patients had delayed detection times in the left upper quadrant in three of four and in the left lower quadrant in two of four VR tasks. A prior VR case study reported that a USN patient’s reaction times were especially delayed in the proximal space and left lower area (Numao et al., 2021). USN related deficits especially in the left lower quadrant have also been demonstrated in non-VR studies presenting targets in peripersonal space (Pitzalis et al., 1997, Cazzoli et al., 2011, Andres et al., 2019). In the Storage and Shoot the target tasks, we displayed targets in the extrapersonal space, which may explain partial differences between the studies. Altogether, the sensitivity and specificity of USN assessments can probably be improved by measuring specific temporal and spatial information on a millisecond level across the different spatial domains (Kaiser et al., 2022).

The present study showed variations between the Shoot the target single and multiple tasks concerning total scores and detection times in different spatial locations, which could relate to the differences in the task types or their varying ability to detect specific USN symptoms, First, USN+ patients’ total left score was lower than the controls’ in the single task variant, indicating that USN+ patients made more errors and had more omissions on the left side. Instead, USN+ patients’ total score was significantly lower than the controls’ in the multiple task variant, showing both lateralized and non-lateralized attention deficits. General inattention has been shown to co-occur with USN (Ting et al., 2011, Nurmi et al., 2018, Villarreal et al., 2021). The difference between the score results in single and multiple tasks may also partly relate to the practice effect: a single task was always performed before multiple task. Second, in the single task variant of Shoot the targets, delayed detection times in USN+ patients were evident in most of the different spatial locations, while the multiple task variant revealed more time-related contralesional deficits. This difference may be explained by the requirements of executive control in multitasking (Strobach et al., 2018), which may hinder the use of compensatory strategies and thus expose subtle contralesional symptoms (van Kessel et al., 2013; Andres et al., 2019). The benefits of an additional task demand to reveal USN has been reported in previous computer-based and VR studies (Bonato, 2012; Aravind et al., 2015; Bonato, 2015; Blini et al., 2016; Ogourtsova et al., 2018a; Andres et al., 2019), which should be considered when assessing mild USN patients.

Finally, we showed some significant but mostly low or moderate correlations between traditional USN evaluation tests and VR task scores. This may indicate that poor performance on traditional USN evaluation tests is associated with impaired performance in VR tasks. Variables related to bilateral performance in the Extinction task and gaze asymmetry in the Storage task with objects were most robustly correlated with performance on traditional paper-and-pencil USN evaluations tests. Fordell et al. (2011) similarly found a strong correlation between the BIT neuropsychological test battery and their VR extinction task that was presented in the peripersonal space. In our study, the modest correlations in some tests might relate to the qualitative differences in the outcome variables presented here. Many of the VR task variables used in our study are based on measuring response times while traditional USN evaluation tests often measure amount of the correct targets or omissions. Another recent study (Ogourtsova et al., 2018a) using partly similar USN evaluation methods (LBT, SCT, Apples test) did not find any correlation with traditional USN evaluation tests and detection time (s) in their VR detection task. Overall, additional research with larger sample are needed to assess validity of these VR tasks and their usefulness in clinical settings in conjunction with traditional USN evaluation methods.

Over half of the participants (52%) reported that they had used VR goggles before. However, there was no difference in previous VR experience between the groups (USN+ patients, USN− patients, and controls). There was also no evidence on the effect of previous VR experience on VR task performance in this study. Hence, it seems that the prior VR experience does not influence any major conclusions derived from the results. We presume that the exposure effects are lacking in this study because the VR-tasks presented in the manuscript are very easy to acquire and intuitive. However, this issue should be verified in a larger sample enabling the use of parametric tests such as ANOVA/ANCOVA.

Our preliminary findings expand on those of previous USN related VR studies (Fordell et al., 2011; Aravind and Lamontagne, 2014; Ogourtsova et al., 2018a; Knobel et al., 2020; Yasuda et al., 2020; Chen and Krch, 2022), indicating minimal reported adverse effects. The ratings of feasibility and acceptance were also high, as demonstrated before in one fully immersive VR study assessing USN (Knobel et al., 2020). Usability in our VR assessment was actually rated very high compared to another USN related VR study that used computer, monitor, a robotic pen and eye shutter stereoscopic glasses to create an immersive 3D experience (Fordell et al., 2011). This finding is aligned with a recent paper showing that stroke patients’ user-experience (i.e., feeling of engagement and presence) was higher when tested with immersive VR by using HDM compared to non-immersive VR by using a computer monitor (Spreij et al., 2022). To our knowledge, this was the first study with USN patients that confirmed a high usability of fully immersive VR tasks when also combined with an eye-tracking system. These preliminary results indicate VR to be safe to use even in a very early stage of stroke recovery. VR tasks can be performed at bedside, and thus VR task battery presented here could potentially be utilized early on neuropsychological assessment or rehabilitation, even with patients who have a severe hemiparesis or hemiplegia. However, to confirm these promising results concerning feasibility and acceptance, larger number of participants are needed in future studies.

Even though immersive virtual reality (VR) may bring several advantages to clinical neuropsychology, there are some issues in the VR use for stroke patients’ assessment. For example, some stroke patients may have dysfunction in the vestibular or oculomotor systems and thus may be especially susceptible to experience cybersickness (Kourtesis et al., 2021). Cybersickness is a form of visually induced motion sickness producing various negative symptoms like nausea or dizziness during or following VR exposure (Weech et al., 2019; Saredakis et al., 2020). Cybersickness has been proposed to manifest from several different reasons (i.e., used VR technology and content, mismatches between observed and expected sensory signals, prolonged use of VR interfaces) and vary across users (Kennedy et al., 2010; Martirosov and Kopecek, 2017; Weech et al., 2019). Moreover, stroke severity and thus stroke related cognitive, motor or sensory impairments may have an impact on the patients’ VR and traditional task performance. In our pilot study, no one reported symptoms of cybersickness and stroke patients had mostly mild neurological and cognitive symptoms when assessed by NIHSS and MoCA. We also carefully evaluated whether participation would be possible, and we excluded patients with interfering impairments (e.g., medically unstable condition, severe aphasia or other conditions significantly impairing cooperation). All the VR-tasks were performed with gaze or verbally, so potential stroke related motor impairments most probably do not have an effect to our VR-task results. Furthermore, most of our participants had right-hand dominance so they were able to successfully use a pencil with their non-affected dominant hand in the USN evaluation test and other neuropsychological paper-and-pencil tasks.

Overall, these results should be interpreted with caution due to the small sample size. Moreover, the observations of this study are limited to patients with mild visual USN and neurological symptoms and should therefore not be generalized to patients with other USN variations or degrees of difficulty. In future studies, it would be important to evaluate how patients with severe USN are able to perform on the VR tasks and what is the user-experience and feasibility in this particular patient group. Concerning the statistical analyses of our study, a possibility of false positives exists due to multiple testing of different aspects of similar underlying phenomena. However, the overarching pattern across all our results clearly points to an effect of USN. We can neither exclude the possibility that the USN− patient group includes patients with latent or very subtle USN symptoms, affecting the results. Principally, USN was diagnosed by cut-offs of BIT subtasks, the Bells test, and CBS self-evaluation form. The sensitivity of traditional tests in assessing mild USN may be weak (Williams et al., 2021). In the future, the results would need replicating with a larger sample size including also more healthy controls to obtain normative data.

The strength of this study is that we used a wide task battery with new and exact VR measures and assessed usability of the VR system. Contrary to other USN related VR assessment studies (described in detail in reviews; Tsirlin et al., 2009; Pedroli et al., 2015; Ogourtsova et al., 2017; Cavedoni et al., 2022), we used gaze-based responses and thus excluded the possible distorting effect of motor action on task performance. Prior studies have shown that response mode may affect to the USN manifestation (Pegna et al., 2001), and gaze-based pointing is faster than hand-based (Adhanom et al., 2023). We also focused on a homogenous group of patients with first-ever right hemisphere ischaemic stroke and used two control groups: healthy participants and stroke patients without USN.

To conclude, our preliminary findings suggest that VR tasks can be used to reveal various aspects of visual attention deficits associated with mild USN. Furthermore, the VR set-up is user-friendly in an acute stroke setting. Detection of USN, even when subtle, is clinically relevant and may advance individually tailored treatment approaches (Kaiser et al., 2022). Our next plan is to explore stroke patients’ recovery using the same VR task battery and additional functional VR task (Merzon et al., 2022; Seesjarvi et al., 2022). We want to further evaluate typical USN behavior, like exploration strategies, scanning patterns, and initial gaze behavior in naturalistic situations. Eye-tracking technology integrated into VR displays opens remarkable new opportunities to study visual attention in stroke patients, may improve ecological validity of USN evaluation and provide sensitive measures of USN that are not accessible in traditional clinical assessment methods (Clay et al., 2019; Kaiser et al., 2022).

## Data availability statement

The datasets presented in this article are not readily available because privacy and/or ethical restrictions. Individual requests for sharing the data will be considered after additional approval for sharing by the local ethics committee. Requests to access the datasets should be directed to JU, jenni.m.uimonen@helsinki.fi.

## Ethics statement

The studies involving humans were approved by the Ethics Committee of Helsinki University Hospital. The studies were conducted in accordance with the local legislation and institutional requirements. The participants provided their written informed consent to participate in this study.

## Author contributions

JU: Conceptualization, Data curation, Formal analysis, Funding acquisition, Investigation, Methodology, Resources, Writing – original draft. SV: Conceptualization, Methodology, Writing – review & editing. SL: Conceptualization, Data curation, Methodology, Writing – review & editing. AA: Conceptualization, Methodology, Writing – review & editing. PI: Investigation, Methodology, Data curation, Writing – review & editing. JS: Conceptualization, Supervision, Writing – review & editing. MH: Conceptualization, Methodology, Funding acquisition, Project administration, Supervision, Writing – review & editing.
