# Supplementary material for: Virtual reality tasks with eye tracking for mild spatial neglect assessment: a pilot study with acute stroke patients
Source: Front Psychol. 2024 Jan 29;15:1319944. doi: 10.3389/fpsyg.2024.1319944 (PMC10860750; doi:10.3389/fpsyg.2024.1319944)
Supplement: Supplementary file 2 [file Table_2.docx]

Supplementary table 2. Technical parameters of the virtual tasks

| Task name | Targets and distractors | Additional details of the tasks | Parameters |
| --- | --- | --- | --- |
| The Extinction task | Targets pop up at an angle of approximately 30° horizontally and 15° vertically. 48 targets appear in the task; 16 on the left side, 16 on the right side, and 16 on both sides. 24 targets appear on personal space (0.7 m from the participant), and 24 targets on extrapersonal space (2.5 m from the participant). 24 targets appear on the upper and lower parts. The targets’ absolute size is always the same despite location. Target duration varies between 100 – 300 ms. Time between targets varies from 3 to 5 s. No distractors were used to assess fundamental extinction characteristics in 3D space. | Study group comprised only stroke patients with right hemisphere stroke. We wanted to maximize task sensitivity between the left USN+ and USN– patients; therefore, the left side targets’ duration was shortest (100 ms). The right-side targets’ duration was 200 ms; the bilateral targets’ duration was 300 ms. | 1. Correct unilateral and bilateral responses in different locations 2. Bilateral omissions in different locations. Omissions for the bilateral target(s) were defined as follows: If the participant incorrectly reported a target location as being “right” when it actually appeared bilaterally, the response was interpreted as “left omission.” The response was similarly interpreted as “right omission” if the target was reported as “left” when it actually appeared bilaterally. |
| The Storage task with objects and figures | Items are located at an angle of approximately 32° horizontally and 18° vertically in both tasks. The Storage task with objects includes objects used in everyday life, and the Storage task with figures has nine different 3D figures (cube, sphere, cylinder, tetrahedron, heart, octahedron, torus, star, octagon) in four different colors (blue, green, yellow, red). Both tasks contain 36 items. Participants search for 24 targets (12 from left side and 12 from right side) in both tasks. The other 12 objects and figures are static distractors. | The storage shelving includes 4 tiers and is divided by location into 10 columns (1 - 10 starting from left) and 4 rows (1 - 4 starting from up). We defined columns 1 - 2 as extreme left, 3 - 4 as middle left, 5 - 6 as middle (no targets appeared in this area), 7 - 8 as middle right, and 9 - 10 as extreme right. We defined rows 1 - 2 as upper parts and rows 3 - 4 as lower parts. The task was planned so three targets appear in all columns except the middle. Additionally, targets appear evenly in the upper and lower parts (12 targets in rows 1 - 2, and 12 targets in rows 3 - 4). | 1. Total search time (includes reading and selection time of the target)  2. Time from target appearance to detection in different locations (excluding reading time of the target). Response window was 15 s from target onset, after which the target automatically disappeared and the next one would appear 3. Omissions in different locations  4. Incorrect target selection 5. Total head movement (m) 6. Gaze asymmetry score [gaze duration (%) on the left side divided by the gaze duration (%) on the right side]  7. Gaze duration (%) in different locations  We were forced to exclude incorrect target(s) from the analyses concerning "detection time" and "gaze duration (%)" because they included selection time (2.2 s). That would have distorted the results. |
| Shoot the target single and multiple tasks | **Single task**: Targets and distractors pop up at an angle of approximately 30° horizontally and 18° vertically. Participants search for one target at a time in single task. The other figures seen concurrently in the visual space are distractors. The correct target type changes every 30 s. The number of targets (40 - 48) and distractors (24 - 32) in every quadrant slightly varied between the participants because of that or by participants´ response speed.  **Multiple task**: Targets and distractors pop up at an angle of approximately 40° horizontally and 18° vertically. Participants search for two targets concurrently in multiple task. The other figures seen concurrently in the visual space are distractors. Correct target types change every 30 s. The number of targets (42 – 49) and distractors (23 – 30) in every quadrant slightly varied between the participants because of that or by participants´ response speed. | In the single and multiple task targets and distractors appear randomly at 3 - 4 meters distance from participants in both task versions, and their lifetime is 7 seconds maximum. New target/ distractors appear every second; however, the task is planned so that a maximum of 5 targets/ distractors are visible simultaneously. Altogether, 72 items (targets or distractors) appear in the task. Targets/ distractors appear in different colors (orange, red, pink, green, yellow, blue) and in four different shapes (heart, diamond, star, octagon) in both task versions. All targets and distractors spin in place in the visual space. | 1. Total search time 2. Time from target appearance to detection in different locations  3. Total score (correctly selected targets divided by the number of presented targets + incorrectly chosen targets)  4. Total score left (correctly selected targets on left side divided by the number of presented targets + incorrectly chosen targets on the left side)  5. Total score right (correctly selected targets on right side divided by the number of presented targets + incorrectly chosen targets on the right side)  6. Incorrect target selection |
