# Supplementary material for: Virtual reality tasks with eye tracking for mild spatial neglect assessment: a pilot study with acute stroke patients
Source: Front Psychol. 2024 Jan 29;15:1319944. doi: 10.3389/fpsyg.2024.1319944 (PMC10860750; doi:10.3389/fpsyg.2024.1319944)
Supplement: Supplementary file 3 [file Table_3.DOCX]

| Supplementary table 3. Detection times in the Storage subtask with figures | | | | | |  |  |  |  |
| --- | --- | --- | --- | --- | --- | --- | --- | --- | --- |
| Variables | | | USN+ (n=4)^e^ | USN– (n=6) | Controls (n=10) | χ2/U | df | *p* | Effect size^d^ |
| Total search time (s) ^a, b^ | | | 141 (12) | 114 (20) | 116 (19) | 8.093 | 2 | .017 | η2=.358*** |
|  | Post hoc comparisons ^c^ | |  |  |  |  |  |  |  |
|  |  | USN+ vs. USN– |  |  |  | 0 |  | .033 | r= .809*** |
|  |  | USN– vs. C |  |  |  | 28.000 |  | 1.000 |  |
|  |  | USN+ vs. C |  |  |  | 2.000 |  | .033 | r= .680*** |
| Detection time total (ms) ^a, b^ | | | 2914 (718) | 1740 (722) | 1862 (574) | 7.583 | 2 | .023 | η2=.328*** |
|  | Post hoc comparisons ^c^ | |  |  |  |  |  |  |  |
|  |  | USN+ vs. USN– |  |  |  | 0 |  | .033 | r= .809*** |
|  |  | USN– vs. C |  |  |  | 28.000 |  | 1.000 |  |
|  |  | USN+ vs. C |  |  |  | 3.000 |  | .048 | r= .643*** |
| Detection time left (ms) ^a, b^ | | | 3432 (1400) | 1816 (349) | 1770 (600) | 8.600 | 2 | .014 | η2=.388*** |
|  | Post hoc comparisons ^c^ | |  |  |  |  |  |  |  |
|  |  | USN+ vs. USN– |  |  |  | 0 |  | .033 | r= .809*** |
|  |  | USN– vs. C |  |  |  | 28.000 |  | 1.000 |  |
|  |  | USN+ vs. C |  |  |  | 1.000 |  | .021 | r= .718*** |
| Detection time right (ms) ^a, b^ | | | 2313 (202) | 1723 (1023) | 1903 (864) | 2.743 | 2 | .254 |  |
| Detection time extreme left (ms) ^a, b^ | | | 3420 (2057) | 1858 (740) | 1594 (391) | 8.116 | 2 | .017 | η2=.360*** |
|  | Post hoc comparisons ^c^ | |  |  |  |  |  |  |  |
|  |  | USN+ vs. USN– |  |  |  | 0 |  | .033 | r= .809*** |
|  |  | USN– vs. C |  |  |  | 26.000 |  | 1.000 |  |
|  |  | USN+ vs. C |  |  |  | 2.000 |  | .033 | r= .680*** |
| Detection time middle left (ms) ^a, b^ | | | 3287 (1242) | 1699 (634) | 1742 (1145) | 6.572 | 2 | .037 | η2=.269*** |
|  | Post hoc comparisons ^c^ | |  |  |  |  |  |  |  |
|  |  | USN+ vs. USN– |  |  |  | 1.000 |  | .057 |  |
|  |  | USN– vs. C |  |  |  | 28.000 |  | 1.000 |  |
|  |  | USN+ vs. C |  |  |  | 4.000 |  | .072 |  |
| Detection time extreme right (ms) ^a, b^ | | | 2789 (479) | 1947 (996) | 2264 (1165) | 2.931 | 2 | .231 |  |
| Detection time middle right (ms) ^a, b^ | | | 1692 (591) | 1489 (1033) | 1394 (1166) | 1.477 | 2 | .478 |  |
| Detection time upper parts (ms) ^a, b^ | | | 3230 (1424) | 1691 (880) | 1904 (937) | 7.154 | 2 | .028 | η2=.303*** |
|  | Post hoc comparisons ^c^ | |  |  |  |  |  |  |  |
|  |  | USN+ vs. USN– |  |  |  | 1.000 |  | .057 |  |
|  |  | USN– vs. C |  |  |  | 26.000 |  | 1.000 |  |
|  |  | USN+ vs. C |  |  |  | 3.000 |  | .048 | r= .643*** |
| Detection time lower parts (ms) ^a, b^ | | | 2406 (395) | 1895 (735) | 1656 (721) | 5.595 | 2 | .061 |  |
| Detection time left upper parts (ms) ^a, b^ | | | 3603 (1885) | 1677 (700) | 1717 (817) | 8.246 | 2 | .016 | η2=.367*** |
|  | Post hoc comparisons ^c^ | |  |  |  |  |  |  |  |
|  |  | USN+ vs. USN– |  |  |  | 1.000 |  | .057 |  |
|  |  | USN– vs. C |  |  |  | 25.000 |  | 1.000 |  |
|  |  | USN+ vs. C |  |  |  | 1.000 |  | .021 | r= .718*** |
| Detection time left lower parts (ms) ^a, b^ | | | 3189 (987) | 1868 (350) | 1803 (880) | 8.048 | 2 | .018 | η2=.356*** |
|  | Post hoc comparisons ^c^ | |  |  |  |  |  |  |  |
|  |  | USN+ vs. USN– |  |  |  | 0 |  | .033 | r= .809*** |
|  |  | USN– vs. C |  |  |  | 28.000 |  | 1.000 |  |
|  |  | USN+ vs. C |  |  |  | 2.000 |  | .033 | r= .680*** |
| Detection time right upper parts (ms) ^a, b^ | | | 2856 (963) | 1803 (1002) | 2131 (1124) | 5.393 | 2 | .067 |  |
| Detection time right lower parts (ms) ^a, b^ | | | 1936 (893) | 1854 (1028) | 1520 (1021) | .155 | 2 | .926 |  |
| Abbreviations: Unilateral spatial neglect, USN; Patients with USN, USN+; Patients without USN, USN–; Controls, C | | | | | | | | | |
| ^a^Median (Interquartile range) | | |  |  |  |  |  |  |  |
| ^b^ p values were calculated by Kruskal-Wallis test (χ2) | | | | |  |  |  |  |  |
| ^c^ Mann-Whitney U-test was used for multiple pairwise comparisons, p values adjusted by the Bonferroni correction | | | | | | | | | |
| ^d^ Effect sizes according to Cohen, 1988: η2 = *small >.01, **medium >.06, ***large >.14 and r = *small >.1, **medium >.3, ***large >.5 | | | | | | | | | |
| ^e^ One USN+ patient needed to be excluded from the analysis because problems of understanding the idea in the task | | | | | | | | | |
