# Supplementary material for: Virtual reality tasks with eye tracking for mild spatial neglect assessment: a pilot study with acute stroke patients
Source: Front Psychol. 2024 Jan 29;15:1319944. doi: 10.3389/fpsyg.2024.1319944 (PMC10860750; doi:10.3389/fpsyg.2024.1319944)
Supplement: Supplementary file 4 [file Table_4.DOCX]

| Supplementary table 4. Task performance in the Storage subtask with figures | | | | | | |  |  |  |
| --- | --- | --- | --- | --- | --- | --- | --- | --- | --- |
| Variables | | | USN+ (n=4)^e^ | USN– (n=6) | Controls (n=10) | χ2/U | df | *p* | Effect size^d^ |
| Omissions total ^a, b^ | | | 0 (0) | 0 (0) | 0 (0) | 0.000 | 2 | 1.000 |  |
| Omissions left ^a, b^ | | | 0 (0) | 0 (0) | 0 (0) | 0.000 | 2 | 1.000 |  |
| Omissions right ^a, b^ | | | 0 (0) | 0 (0) | 0 (0) | 0.000 | 2 | 1.000 |  |
| Incorrect target selection ^a, b^ | | | 0 (0) | 0 (0) | 0 (0) | 0.704 | 2 | .703 |  |
| Total head movement when playing (m) ^a, b^ | | | 2.0 (4.8) | 3.2 (2.3) | 2.5 (1.2) | .509 | 2 | .775 |  |
| Gaze asymmetry score left/right ^a, b^ | | | 0.9 (0.2) | 1.1 (.5) | 1 (0.2) | 4.379 | 2 | .112 |  |
| Gaze duration in left (%) ^a, b^ | | | 44.4 (5) | 49.9 (10.8) | 47.7 (5.3) | 6.036 | 2 | .049 | η2=.237*** |
|  | Post hoc comparisons ^c^ | |  |  |  |  |  |  |  |
|  |  | USN+ vs. USN– |  |  |  | 6.000 |  | .603 |  |
|  |  | USN– vs. C |  |  |  | 26.000 |  | 1.000 |  |
|  |  | USN+ vs. C |  |  |  | 0.000 |  | .015 | r= .756*** |
| Gaze duration in right (%) ^a, b^ | | | 50 (5.1) | 44.4 (9.9) | 46.6 (4.8) | 2.973 | 2 | .226 |  |
| Gaze duration in extreme left (%) ^a, b^ | | | 16.9 (8.5) | 20.6 (3.9) | 18.7 (3.6) | 3.573 | 2 | .168 |  |
| Gaze duration in middle left (%) ^a, b^ | | | 27.5 (3.6) | 28.4 (6.8) | 29 (4.5) | 1.467 | 2 | .480 |  |
| Gaze duration in middle right (%) ^a, b^ | | | 29.9 (7.7) | 25.6 (5.5) | 28.4 (3.1) | 1.166 | 2 | .558 |  |
| Gaze duration in extreme right (%) ^a, b^ | | | 21.6 (4.5) | 19.3 (4.5) | 19 (1.8) | 2.061 | 2 | .357 |  |
| Abbreviations: Unilateral spatial neglect, USN; Patients with USN, USN+; Patients without USN, USN–; Controls, C | | | | | | | | | |
| ^a^Median (Interquartile range) | | |  |  |  |  |  |  |  |
| ^b^ p values were calculated by Kruskal-Wallis test (χ2) | | | |  |  |  |  |  |  |
| ^c^ Mann-Whitney U-test was used for multiple pairwise comparisons, p values adjusted by the Bonferroni correction | | | | | | | | | |
| ^d^ Effect sizes according to Cohen, 1988: η2 = *small >.01, **medium >.06, ***large >.14 and r = *small >.1, **medium >.3, ***large >.5 | | | | | | | | | |
| ^e^ One USN+ patient needed to be excluded from the analysis because problems of understanding the idea in the task | | | | | | | | | |
