# Supplementary material for: Virtual reality tasks with eye tracking for mild spatial neglect assessment: a pilot study with acute stroke patients
Source: Front Psychol. 2024 Jan 29;15:1319944. doi: 10.3389/fpsyg.2024.1319944 (PMC10860750; doi:10.3389/fpsyg.2024.1319944)
Supplement: Supplementary file 5 [file Table_5.DOCX]

| Supplementary table 5. Detection times in the Shoot the target multiple task | | | | | | |  |  |  |
| --- | --- | --- | --- | --- | --- | --- | --- | --- | --- |
| Variables | | | USN+ (n=5) | USN– (n=6) | Controls (n=10) | χ2/U | df | *p* | Effect size^d^ |
| Total search time (s) ^a, b^ | | | 122 (6) | 117 (13) | 115 (6) | 4.960 | 2 | .084 |  |
| Detection time total (ms) ^a, b^ | | | 3190 (1035) | 2825 (1275) | 2420 (603) | 5.268 | 2 | .072 |  |
| Detection time left (ms) ^a, b^ | | | 3270 (1305) | 2635 (1298) | 2375 (785) | 6.840 | 2 | .033 | η2=.269*** |
|  | Post hoc comparisons ^c^ | |  |  |  |  |  |  |  |
|  |  | USN+ vs. USN– |  |  |  | 8.000 |  | .603 |  |
|  |  | USN– vs. C |  |  |  | 20.000 |  | .834 |  |
|  |  | USN+ vs. C |  |  |  | 3.500 |  | .024 | r= .681*** |
| Detection time right (ms) ^a, b^ | | | 3300 (760) | 2965 (1313) | 2740 (768) | 2.758 | 2 | .252 |  |
| Detection time upper quadrants (ms) ^a, b^ | | | 3580 (1225) | 2875 (1140) | 2585 (828) | 7.362 | 2 | .025 | η2=.298*** |
|  | Post hoc comparisons ^c^ | |  |  |  |  |  |  |  |
|  |  | USN+ vs. USN– |  |  |  | 6.000 |  | .300 |  |
|  |  | USN– vs. C |  |  |  | 19.000 |  | .696 |  |
|  |  | USN+ vs. C |  |  |  | 4.000 |  | .030 | r= .664*** |
| Detection time lower quadrants (ms) ^a, b^ | | | 2820 (1220) | 2705 (1458) | 2300 (848) | 2.049 | 2 | .359 |  |
| Detection time upper left (ms) ^a, b^ | | | 3630 (1345) | 2660 (805) | 1985 (533) | 8.752 | 2 | .013 | η2=.375*** |
|  | Post hoc comparisons ^c^ | |  |  |  |  |  |  |  |
|  |  | USN+ vs. USN– |  |  |  | 7.000 |  | .432 |  |
|  |  | USN– vs. C |  |  |  | 12.000 |  | .153 |  |
|  |  | USN+ vs. C |  |  |  | 4.000 |  | .030 | r= .664*** |
| Detection time lower left (ms) ^a, b^ | | | 2950 (1560) | 2610 (1613) | 2490 (1250) | 2.171 | 2 | .338 |  |
| Detection time upper right (ms) ^a, b^ | | | 3770 (1295) | 3055 (1543) | 3335 (1163) | 4.108 | 2 | .128 |  |
| Detection time lower right (ms) ^a, b^ | | | 2140 (1370) | 2800 (1118) | 2415 (665) | 1.390 | 2 | .499 |  |
| Abbreviations: Unilateral spatial neglect, USN; Patients with USN, USN+; Patients without USN, USN–; Controls, C | | | | | | | | | |
| ^a^Median (Interquartile range) | | | |  |  |  |  |  |  |
| ^b^ p values were calculated by Kruskal-Wallis test (χ2) | | | | |  |  |  |  |  |
| ^c^ Mann-Whitney U-test was used for multiple pairwise comparisons, p values adjusted by the Bonferroni correction | | | | | | | | | |
| ^d^ Effect sizes according to Cohen, 1988: η2 = *small >.01, **medium >.06, ***large >.14 and r = *small >.1, **medium >.3, ***large >.5 | | | | | | | | | |
