# Supplementary material for: Virtual reality tasks with eye tracking for mild spatial neglect assessment: a pilot study with acute stroke patients
Source: Front Psychol. 2024 Jan 29;15:1319944. doi: 10.3389/fpsyg.2024.1319944 (PMC10860750; doi:10.3389/fpsyg.2024.1319944)
Supplement: Supplementary file 6 [file Table_6.DOCX]

| Supplementary table 6. Task performance in the Shoot the target multiple task | | | | | | | |  |  |
| --- | --- | --- | --- | --- | --- | --- | --- | --- | --- |
| Variables | | | USN+ (n=5) | USN– (n=6) | Controls (n=10) | χ2/U | df | *p* | Effect size^d^ |
| Total score ^a, b^ | | | 0.78 (0.29) | 0.93 (0.12) | 0.96 (0.6) | 9.092 | 2 | .011 | η2=.394*** |
|  | Post hoc comparisons ^c^ | | |  |  |  |  |  |  |
|  |  | USN+ vs. USN– |  |  |  | 5.000 |  | .204 |  |
|  |  | USN– vs. C |  |  |  | 12.500 |  | .171 |  |
|  |  | USN+ vs. C |  |  |  | 4.000 |  | .030 | r= .664*** |
| Total score left ^a, b^ | | | 0.75 (0.33) | 0.92 (0.14) | 0.98 (0.81) | 6.021 | 2 | .049 | η2=.223*** |
|  | Post hoc comparisons ^c^ | | |  |  |  |  |  |  |
|  |  | USN+ vs. USN– |  |  |  | 7.500 |  | .510 |  |
|  |  | USN– vs. C |  |  |  | 17.000 |  | .441 |  |
|  |  | USN+ vs. C |  |  |  | 7.500 |  | .081 |  |
| Total score right ^a, b^ | | | 0.81 (0.23) | 0.88 (0.14) | 0.95 (0.13) | 2.755 | 2 | .252 |  |
| Incorrect target selection ^a, b^ | | | 1 (5) | 1 (2) | 0.5 (1) | .690 | 2 | .708 |  |
| Abbreviations: Unilateral spatial neglect, USN; Patients with USN, USN+; Patients without USN, USN–; Controls, C | | | | | | | | | |
| ^a^Median (Interquartile range) | | | |  |  |  |  |  |  |
| ^b^ p values were calculated by Kruskal-Wallis test (χ2) | | | | | |  |  |  |  |
| ^c^ Mann-Whitney U-test was used for multiple pairwise comparisons, p values adjusted by the Bonferroni correction | | | | | | | | | |
| ^d^ Effect sizes according to Cohen, 1988: η2 = *small >.01, **medium >.06, ***large >.14 and r = *small >.1, **medium >.3, ***large >.5 | | | | | | | | | |
