# Supplementary material for: Virtual reality tasks with eye tracking for mild spatial neglect assessment: a pilot study with acute stroke patients
Source: Front Psychol. 2024 Jan 29;15:1319944. doi: 10.3389/fpsyg.2024.1319944 (PMC10860750; doi:10.3389/fpsyg.2024.1319944)
Supplement: Supplementary file 7 [file Table_7.docx]

| Supplementary table 7. Correlation coefficients (Kendall's tau) between USN evaluation tests and VR tasks results | | | | | | |
| --- | --- | --- | --- | --- | --- | --- |
| Variables | | Bells Test omissions left | LBT  score | LCT  correct targets | SCT  correct targets | SCT  time (s) |
| Extinction | |  |  |  |  |  |
|  | Correct bilateral targets (0-16) | –.106 | **.592**** | .230 | **.592**** | **–.473*** |
|  | Bilateral targets, left omissions (0-16) | .106 | **–.592**** | –.230 | **–.592**** | **.473*** |
| Storage subtask with objects | |  |  |  |  |  |
|  | Total search time (s) | .249 | –.278 | –.221 | –.278 | **.473**** |
|  | Detection time left (ms) | .249 | –.278 | –.341 | –.278 | **.444**** |
|  | Detection time extreme left (ms) | **.371*** | –.247 | –.305 | –.247 | **.454**** |
|  | Gaze asymmetry score left/right | **–.493**** | .247 | **.437*** | .247 | **–.348*** |
| Shoot the target single task | |  |  |  |  |  |
|  | Total search time (s) | **.371*** | –.309 | –.221 | –.309 | .261 |
|  | Detection time total (ms) | **.368*** | –.248 | –.216 | –.248 | .311 |
|  | Detection time left (ms) | **.344*** | –.263 | –.192 | –.263 | **.315*** |
|  | Detection time right (ms) | **.449**** | –.123 | –.197 | –.123 | .251 |
|  | Detection time upper quadrants (ms) | **.394*** | –.309 | –.197 | –.309 | **.338*** |
|  | Detection time upper left (ms) | **.373*** | –.310 | –.234 | –.310 | .291 |
|  | Detection time lower left (ms) | .261 | –.155 | –.138 | –.155 | .228 |
|  | Detection time upper right (ms) | **.461**** | –.247 | –.102 | –.247 | .295 |
|  | Total score left | –.346 | .364 | .515** | .364 | –.330 |
| Shoot the target multiple task | |  |  |  |  |  |
|  | Detection time left (ms) | .206 | –.217 | –.306 | –.217 | **.421**** |
|  | Detection time upper quadrants (ms) | **.373*** | –.279 | **–.355*** | –.279 | .238 |
|  | Detection time upper left (ms) | **.460**** | –.247 | –.221 | –.247 | .261 |
|  | Total score | **–.350*** | .278 | .288 | .278 | **–.363*** |
| Abbreviations: LBT, Line bisection test; SCT, Star cancellation test; LCT, Letter cancellation test. | | | | | | |
| Significant correlations are indicated in bold; p-value * <0.05, ** 0.01 and *** 0.001. | | | | | |  |
